# Supplementary material for: SWR1-Independent Association of H2A.Z to the LINC Complex Promotes Meiotic Chromosome Motion
Source: Front Cell Dev Biol. 2020 Oct 22;8:594092. doi: 10.3389/fcell.2020.594092 (PMC7642583; doi:10.3389/fcell.2020.594092)
Supplement: Supplementary Figure 1 — Genome-wide incorporation of H2A.Z to meiotic chromatin depends on SWR1. (Related to Figure 1) Profiles of H2A.Z binding to all chromosomes in wild type (A), swr1Δ (B), and the untagged control (C), determined by ChIP-seq. Magenta circles indicate the location of the centromere. (D,E) Metagene analysis of H2A.Z binding by ChIP-seq. The ORFs are scaled to the “Start” and “Stop” positions, and up- and downstream flanking regions represent half the size of the ORF. Samples were taken at 0 h and 15 h after meiotic induction. Anti-GFP antibodies were used to immunoprecipitate H2A.Z-GFP. Average profiles from two replicates are shown. Strains are: DP840 (HTZ1-GFP), DP841 (HTZ1-GFP swr1Δ) and DP421 (HTZ1 untagged control). [file Data_Sheet_1.PDF]

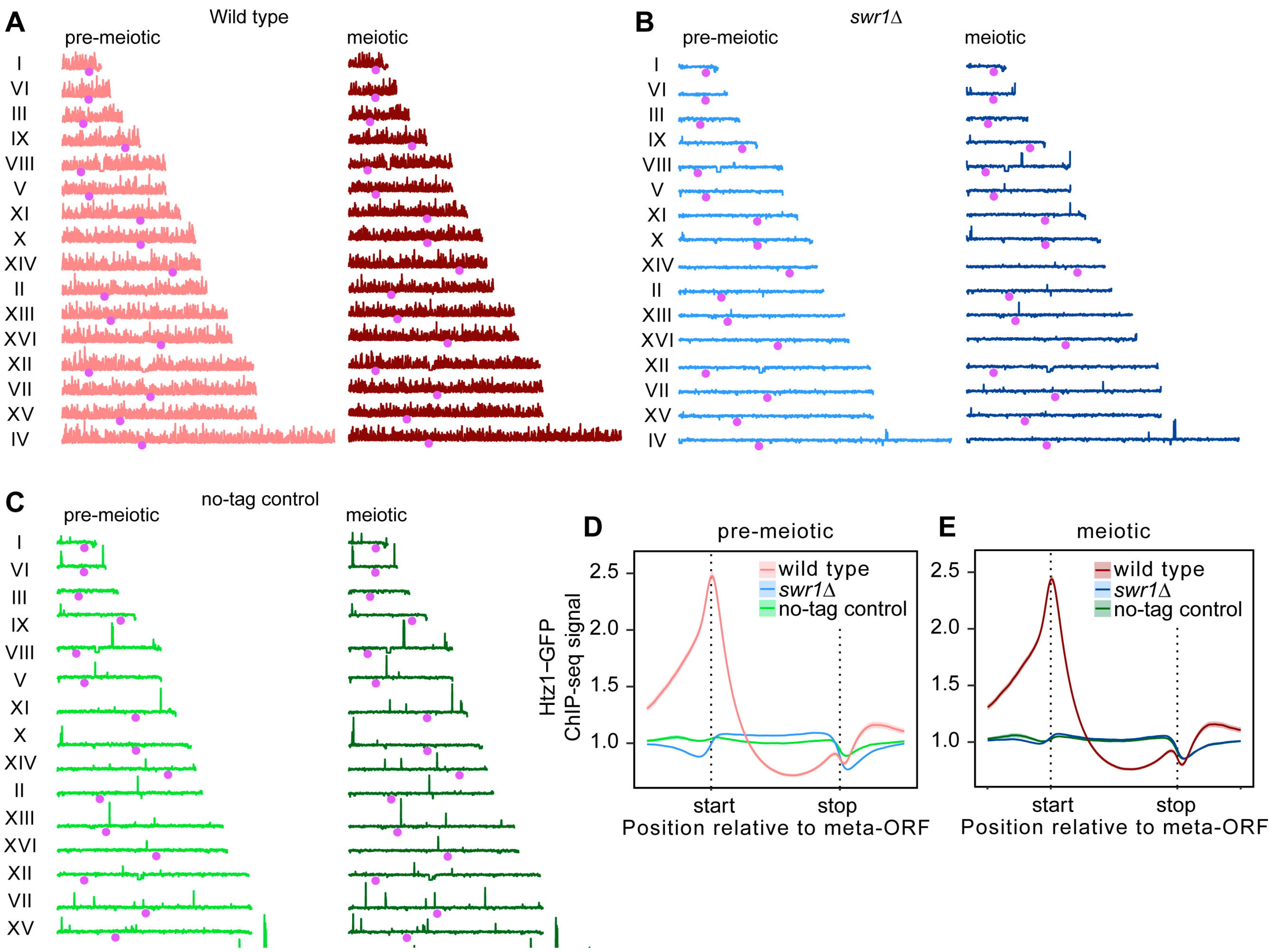

Supplementary Figure 1. Genome-wide incorporation of H2A.Z to meiotic chromatin depends on SWR1. (Related to Figure 1)

Profiles of H2A.Z binding to all chromosomes in wild type **(A)**, *swr1*Δ **(B)**, and the untagged control **(C)**, determined by ChIP-seq. Magenta circles indicate the location of the centromere. **(D-E)** Metagene analysis of H2A.Z binding by ChIP-seq. The ORFs are scaled to the "Start" and "Stop" positions, and up- and downstream flanking regions represent half the size of the ORF. Samples were taken at 0 h and 15 h after meiotic induction. Anti-GFP antibodies were used to immunoprecipitate H2A.Z-GFP. Average profiles from two replicates are shown. Strains are: DP840 (*HTZ1-GFP*), DP841 (*HTZ1-GFP swr1Δ*) and DP421 (*HTZ1* untagged control).

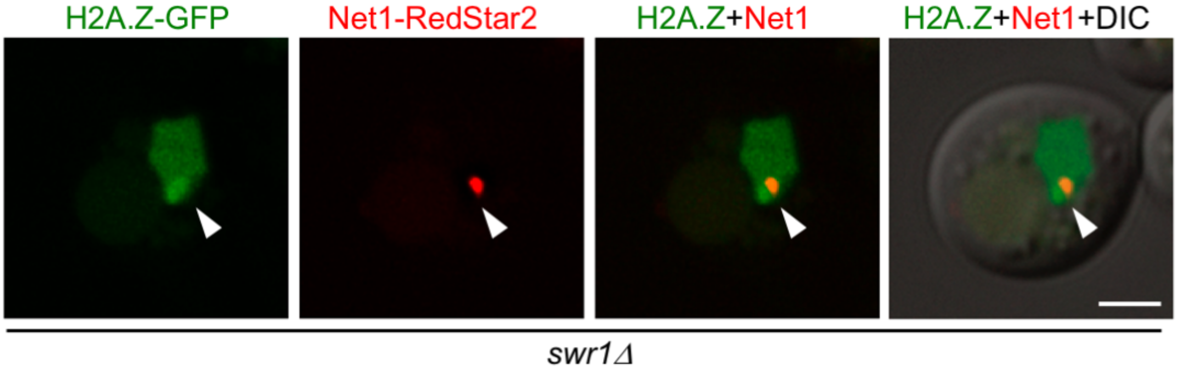

Supplementary Figure 2. A fraction of H2A.Z accumulates in the vicinity of the nucleolus in *swr1Δ* (Related to Figure 2).

Microscopy fluorescence images of *swr1Δ* cells expressing *HTZ1-GFP* and *NET1-RedStar2* as a nucleolar marker. A single plane of a representative cell displaying a diffuse peripheral accumulation of H2A.Z is shown. The arrowhead points to the nucleolar area marked by Net1. Images were taken 16 h after meiotic induction. Scale bar, 2 µm. The strain is DP1189 (*swr1Δ HTZ1-GFP NET1-RedStar2*).

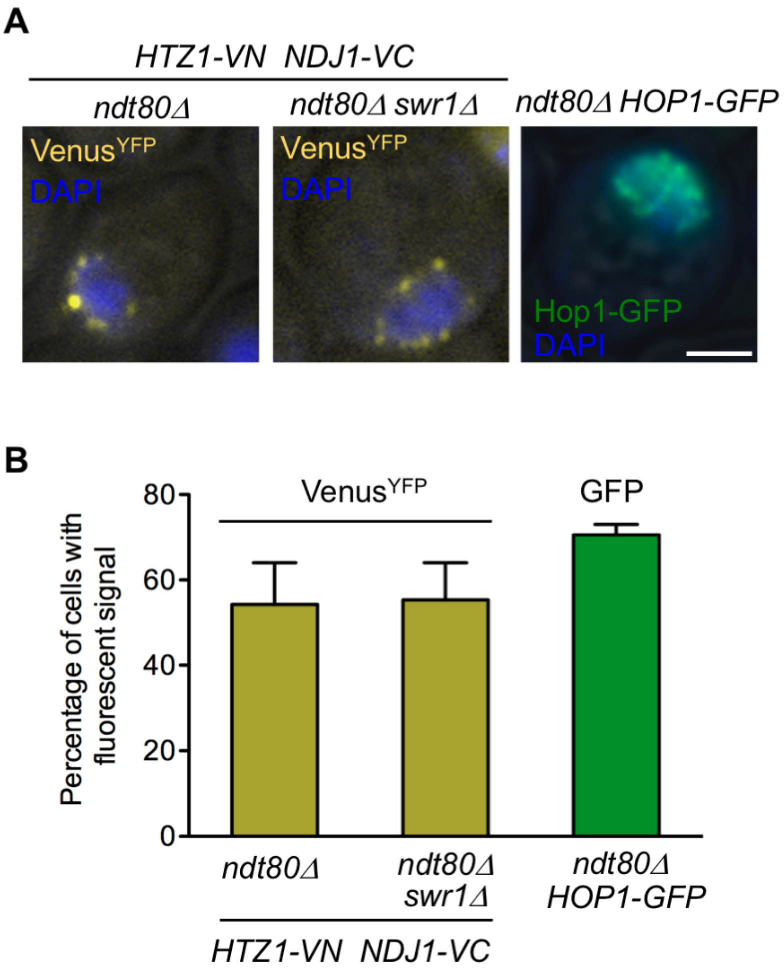

Supplementary Figure 3. BiFC analysis of H2A.Z-Ndj1 interaction in *ndt80Δ* cells (Related to Figure 3).

(A) Microscopy fluorescence images of *ndt80Δ* and *ndt80Δ swr1Δ* cells expressing *HTZ1* fused to the N-terminal half of the Venus^{YFP} (*VN*) and *NDJ1* fused to the C-terminal half of the Venus^{YFP} (*VC*). Nuclei are stained with DAPI (blue). The reconstitution of Venus^{YFP} fluorescence resulting from H2A.Z-VN/Ndj1-VC interaction appears in yellow. A parallel meiotic culture of *ndt80Δ* cells expressing *HOP1-GFP* (green) was used as control for meiotic prophase I staging. Images were taken 24 h after meiotic induction. Representative cells are shown. Scale bar, 2 µm **(B)** Quantification of the percentage of cells displaying Venus^{YFP} fluorescent signal or Hop1-GFP signal, as indicated. The analysis was performed in triplicate. More than 300 cells were scored in every experiment. Error bars, SD. Strains are: DP1748 (*ndt80Δ HTZ1-VN NDJ1-VC*), DP1749 (*ndt80Δ swr1Δ HTZ1-VN NDJ1-VC*) and DP963 (*ndt80Δ HOP1-GFP*).

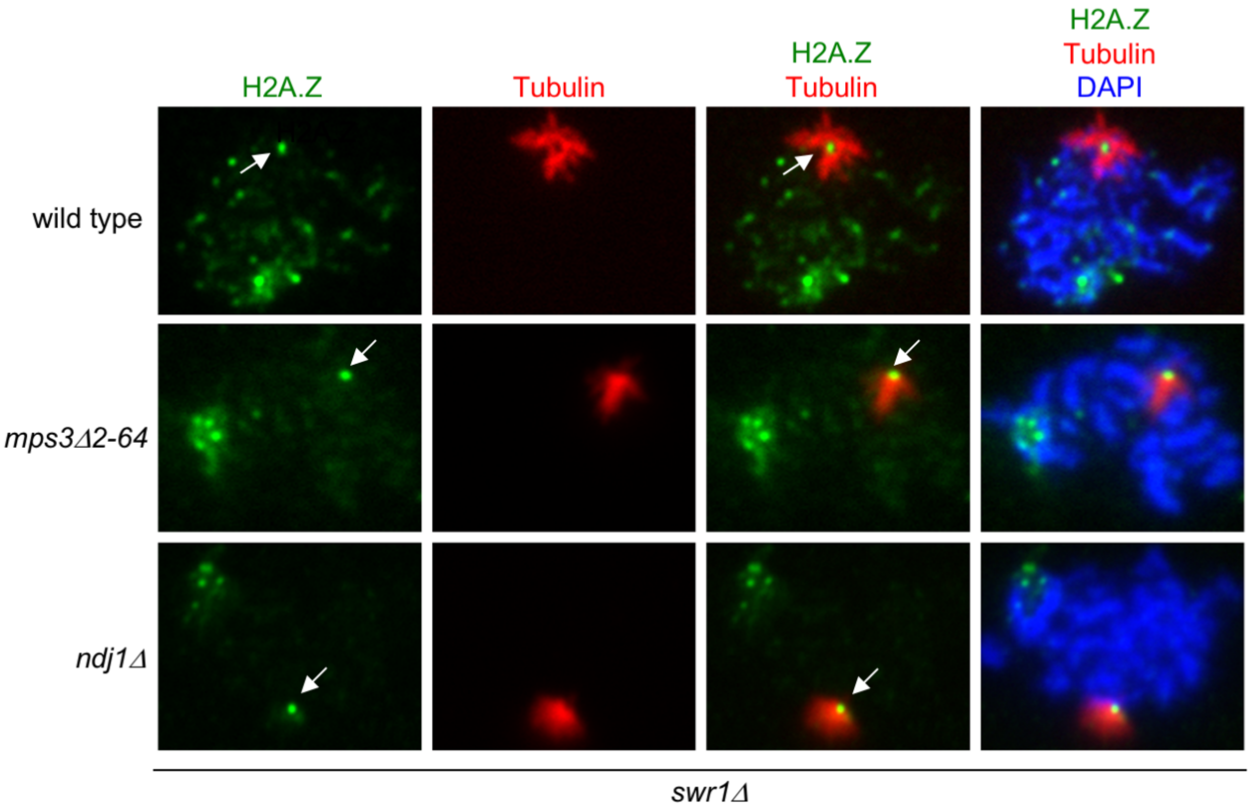

Supplementary Figure 4. Localization of H2A.Z to the SPB is independent of Ndj1 and the 2-64 N-terminal domain of Mps3. (Related to Figure 5)

Immunofluorescence of representative spread pachytene nuclei stained with DAPI to visualize chromatin (blue), anti-GFP to detect H2A.Z (green), and anti-tubulin to mark the monopolar prophase spindle (red). The arrow points to an H2A.Z focus present at the center of the bushy spindle corresponding to the SPB location. Strains are DP1395 (wild type), DP1280 (*mps3-Δ2-64*) and DP1305 (*ndj1Δ*). 25, 21 and 23 nuclei were examined for wild type, *mps3-Δ2-64* and *ndj1Δ*, respectively.

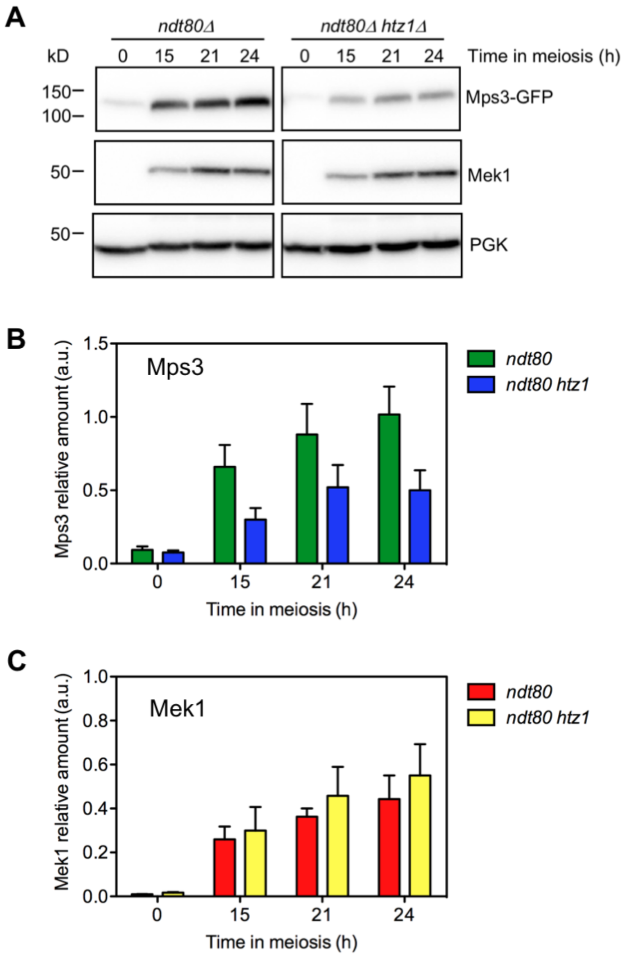

Supplementary Figure 5. Mps3 global levels are reduced in *ndt80Δ*-arrested cells lacking H2A.Z. (Related to Figure 6)

(A) Western blot analysis of Mps3-GFP and Mek1 production during meiosis detected with anti-GFP and anti-Mek1 antibodies, respectively. PGK was used as a loading control. A representative blot is shown. **(B-C)** Quantification of Mps3-GFP (B) and Mek1 (C) levels normalized to PGK. Average and SEM (error bars) from three independent experiments are shown. Strains are: DP1014 (*ndt80Δ MPS3-GFP*) and DP1013 (*ndt80Δ htz1Δ MPS3-GFP*).

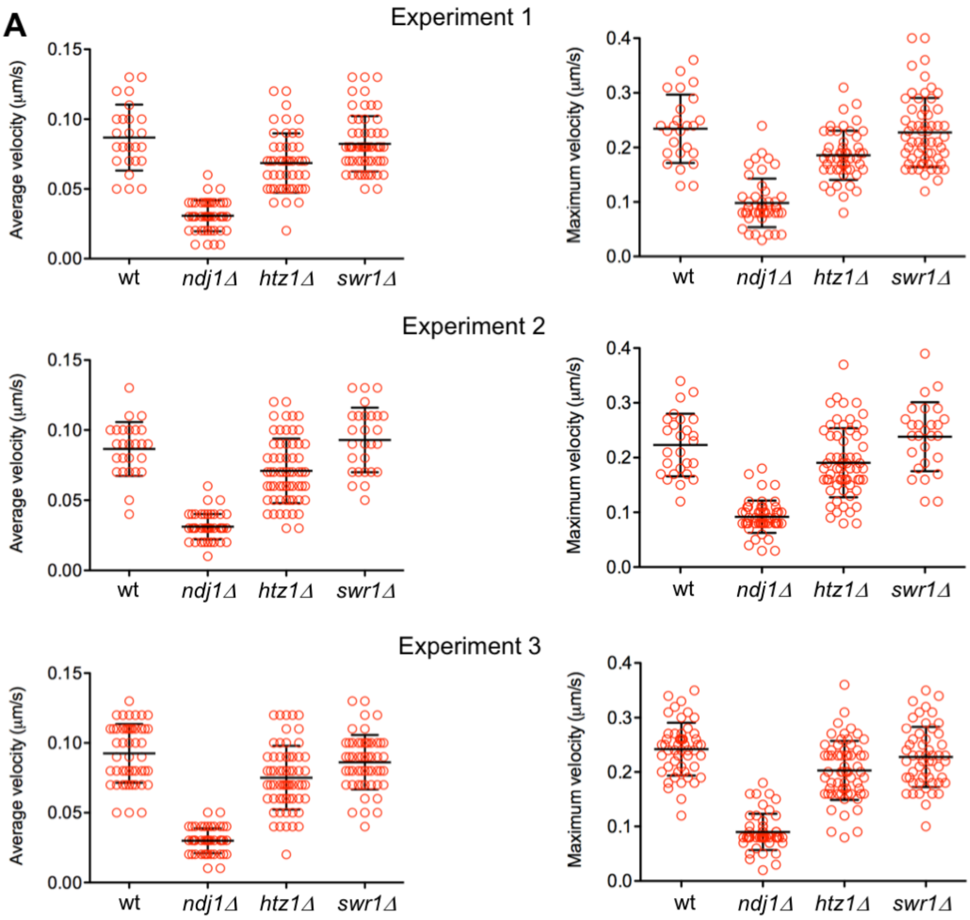

B

Average velocity

ANOVA Tukey. Experiment 1	*ndj1Δ*	*htz1Δ*	*swr1Δ*
wild type	***	***	ns
ndj1Δ		***	***
htz1Δ			**

ANOVA Tukey. Experiment 2	*ndj1Δ*	*htz1Δ*	*swr1Δ*
wild type	***	**	ns
ndj1Δ		***	***
htz1Δ			***

ANOVA Tukey. Experiment 3	*ndj1Δ*	*htz1Δ*	*swr1Δ*
wild type	***	***	ns
ndj1Δ		***	***
htz1Δ			*

ANOVA Tukey. 3 experiments	*ndj1Δ*	*htz1Δ*	*swr1Δ*
wild type	***	***	ns
ndj1Δ		***	***
htz1Δ			**

Maximum velocity

ANOVA Tukey. Experiment 1	*ndj1Δ*	*htz1Δ*	*swr1Δ*
wild type	***	**	ns
ndj1Δ		***	***
htz1Δ			***

ANOVA Tukey. Experiment 2	*ndj1Δ*	*htz1Δ*	*swr1Δ*
wild type	***	ns	ns
ndj1Δ		***	***
htz1Δ			**

ANOVA Tukey. Experiment 3	*ndj1Δ*	*htz1Δ*	*swr1Δ*
wild type	***	***	ns
ndj1Δ		***	***
htz1Δ			*

ANOVA Tukey. 3 experiments	*ndj1Δ*	*htz1Δ*	*swr1Δ*
wild type	***	***	ns
ndj1Δ		***	***
htz1Δ			**

C

		wild type	*ndj1Δ*	*htz1Δ*	*swr1Δ*
**Average velocity (µm/s)**	Experiment 1	0.086 ± 0.023	0.030 ± 0.011	0.068 ± 0.021	0.082 ± 0.020
	Experiment 2	0.087 ± 0.019	0.03 ± 0.009	0.071 ± 0.023	0.092 ± 0.023
	Experiment 3	0.091 ± 0.021	0.029 ± 0.008	0.074 ± 0.023	0.085 ± 0.019
	Mean 3 experiments	0.088 ± 0.003	0.030 ± 0.001	0.071 ± 0.003	0.086 ± 0.005
**Maximum velocity (µm/s)**	Experiment 1	0.234 ± 0.062	0.098 ± 0.046	0.185 ± 0.046	0.227 ± 0.064
	Experiment 2	0.223 ± 0.057	0.091 ± 0.030	0.190 ± 0.064	0.238 ± 0.063
	Experiment 3	0.241 ± 0.048	0.089 ± 0.033	0.202 ± 0.054	0.226 ± 0.055
	Mean 3 experiments	0.232 ± 0.009	0.093 ± 0.005	0.192 ± 0.009	0.230 ± 0.007

Supplementary Figure 6. Analysis of *TEL4L* movement. (Related to Figure 8).
(A) Measurement of average velocity and maximum velocity in three independent time-lapse experiments tracking *TEL4L* movement marked with GFP as depicted in Figure 8. Error bars, SD. **(B)** ANOVA statistical analysis of the velocity data obtained in every individual experiment as well as combining the data from all three experiments. **(C)** Mean values for average and maximum velocity. Strains are DP1692 (wild type), DP1722 (*ndj1Δ*), DP1693 (*htz1Δ*) and DP1694 (*swr1Δ*).

Supplementary Table 1. ***Saccharomyces cerevisiae*** **strains**

Strain	Genotype	Source
DP421	*MAT***a***/MATα leu2,3-112 his4-260 thr1-4 ura3-1 trp1-289 ade2-1 lys2ΔNheI*	PSS Lab
DP838	DP421 *htz1::URA3 ZIP1-GFP*	This work
DP840	DP421 *HTZ1-GFP::kanMX6*	PSS Lab
DP841	DP421 *swr1::natMX4 HTZ1-GFP::kanMX6*	PSS Lab
DP866	DP421 *MPS3-GFP::kanMX6*	This work
DP867	DP421 *htz1::URA3 MPS3-GFP::kanMX6*	This work
DP957	DP421 *ndj1::natMX4 ZIP1-GFP*	This work
DP963	DP421 *ndt80::LEU2 HOP1-GFP::kanMX6*	PSS Lab
DP1013	DP421 *ndt80:LEU2 htz1::URA3 MPS3-GFP::kanMX6*	This work
DP1014	DP421 *ndt80:LEU2 MPS3-GFP::kanMX6*	This work
DP1032	DP421 *MPS3-GFP::kanMX6 HOP1-mCherry::natMX4*	This work
DP1033	DP421 *htz1::URA3 MPS3-GFP::kanMX6 HOP1-mCherry::natMX4*	This work
DP1057	DP421 *ZIP1-GFP PMA1-mCherry::natMX4*	This work
DP1091	DP421 *swr1::natMX4 ZIP1-GFP*	This work
DP1102	DP421 *swr1::natMX4 MPS3-GFP::kanMX6*	This work
DP1103	DP421 *ndj1::natMX4 MPS3-GFP::kanMX6*	This work
DP1108	DP421 *swr1::natMX4 HTZ1-GFP::kanMX6 MPS3-mCherry::hphMX4*	This work
DP1172	DP421 *HTZ1-GFP::kanMX6 CNM67-mCherry::natMX4 swr1::hphMX4*	This work
DP1182	DP421 *HTZ1-GFP::kanMX6 swr1::hphMX4*	This work
DP1189	DP421 *HTZ1-GFP::kanMX6 NET1-RedStar2::natNT2 swr1::hphMX4*	This work
DP1280	DP421 *mps3::hphMX4 pSS326 [mps3Δ2-64-mCherry URA3] HTZ1-GFP::kanMX6 swr1::natMX4*	This work
DP1305	DP421 *HTZ1-GFP::kanMX6 swr1::natMX4 ndj1::hphMX4*	This work
DP1330	DP421 *MPS3-3HA::kanMX6*	This work

DP1394	DP421 *MPS3-3HA::natMX4 HTZ1-GFP::kanMX6*	This work
DP1395	DP421 *swr1::hphMX4 MPS3-3HA::natMX4 HTZ1-GFP::kanMX6*	This work
DP1493	DP421 *swr1::hphMX4 HTZ1-VN::TRP1 NDJ1-VC::kanMX6*	This work
DP1496	DP421 *HTZ1-VN::TRP1 NDJ1-VC::kanMX6*	This work
DP1506	DP421 *SPC110-RedStar2::natNT2/SPC110 HTZ1-VN::TRP1 NDJ1-VC::kanMX6 swr1::hphMX4*	This work
DP1511	DP421 *mps3::natMX4 pSS269 [MPS3-mCherry URA3] HTZ1-VN::TRP1 NDJ1-VC::kanMX6*	This work
DP1512	DP421 *mps3::natMX4 pSS326 [mps3Δ2-64-mCherry URA3] HTZ1-VN::TRP1 NDJ1-VC::kanMX6*	This work
DP1540	DP421 *HTZ1-VN::TRP1*	This work
DP1541	DP421 *NDJ1-VC::kanMX6*	This work
DP1576	DP421 *MPS3-GFP::kanMX6 swr1::natMX4 SPC110-mCherry::hphNT1/SPC110*	This work
DP1578	DP421 *HTZ1-GFP::kanMX6 swr1::natMX4 SPC110-mCherry::hphNT1/SPC110*	This work
DP1692	DP421 *P_{CUP1}-IME1::kanMX6 ZIP1-mCherry/ZIP1 TEL4L-tetO(50)::URA3/TEL4L TetR-GFP::LEU2/leu2*	This work
DP1693	DP421 *htz1::natMX4 P_{CUP1}-IME1::kanMX6 ZIP1-mCherry/ZIP1 TEL4L-tetO(50)::URA3/TEL4L TetR-GFP::LEU2/leu2*	This work
DP1694	DP421 *swr1::hphMX4 P_{CUP1}-IME1::kanMX6 ZIP1-mCherry/ZIP1 TEL4L-tetO(50)::URA3/TEL4L TetR-GFP::LEU2/leu2*	This work
DP1722	DP421 *ndj1::kanMX6 P_{CUP1}-IME1::kanMX6 ZIP1-mCherry/ZIP1 TEL4L-tetO(50)::URA3/TEL4L TetR-GFP::LEU2/leu2*	This work
DP1748	DP421 *ndt80::natMX4 HTZ1-VN::TRP1 NDJ1-VC::kanMX6*	This work
DP1749	DP421 *ndt80::natMX4 swr1::hphMX4 HTZ1-VN::TRP1 NDJ1-VC::kanMX6*	This work

*All strains are diploids isogenic to BR1919 (Rockmill and Roeder, 1990). Unless specified, all strains are homozygous for the indicated markers. DP421 is a *lys2* version of the original BR1919-2N.

Rockmill, B., and G.S. Roeder. 1990. Meiosis in asynaptic yeast. *Genetics*. 126:563-574.

Supplementary Table 2. Plasmids

Plasmid	Vector	Relevant parts	Source/Reference
pSS266	pJET2.1	*MPS3-mCherry*	This work
pSS267	pRS424	*2µ TRP1 MPS3-mCherry*	This work
p SS269	pRS316	*CEN6 URA3 MPS3-mCherry*	This work
pSS326	pRS316	*CEN6 URA3 mps3-2-64Δ-mCherry*	This work
pSS329 (pAC32)	unknown	*tetR-NLS-GFP::LEU2*	Andrés Clemente (IBFG)/ (Michaelis et al., 1997)
pSS330 (pAC18)	pRS406	*TEL4L-tetO(50)::URA3*	Andrés Clemente (IBFG)
pFN21	pFA6a	*mCherry::natMX4*	César Roncero (IBFG)

Michaelis, C., R. Ciosk, and K. Nasmyth. 1997. Cohesins: chromosomal proteins that prevent premature separation of sister chromatids. *Cell*. 91:35-45.

Supplementary Table 3. Primary and secondary antibodies

**Antibody**	**Host and type**	**Application* (Dilution)**	**Source / Reference**
H2A (acidic patch)	Rabbit polyclonal	WB (1:5000)	Merck 07-146
H2A.Z	Rabbit polyclonal	WB (1:1000)	Active Motif 39647
H2B	Rabbit polyclonal	WB (1:5000)	Abcam ab1790
H3	Rabbit polyclonal	WB (1:5000)	Abcam ab1791
H4	Rabbit polyclonal	WB (1:1000)	Abcam ab10158
Zip1	Rabbit polyclonal	IF (1:300)	S. Roeder
HA (12CA5)	Mouse monoclonal	WB (1:1000)	Roche 11666606001
HA (3F10)	Rat monoclonal	IF (1:225)	Roche 11867431001
GFP (JL-8)	Mouse monoclonal	WB (1:1000-1:2000)<br>IF (1:200)	Clontech 632381
GFP	Rabbit polyclonal	ChIP-seq (3μl)	R. Freire
mCherry/DsRed	Rabbit polyclonal	IF (1:200)	Clontech 632496
Pgk1 (22C5D8)	Mouse monoclonal	WB (1:5000)	Invitrogen 459250
Mek1	Rabbit polyclonal	WB (1:1000)	Ontoso et al., 2013
Tubulin	Rabbit monoclonal	IF (1:500)	Abcam EPR13798
Anti-mouse-HRP	Sheep polyclonal	WB (1:5000)	GE-Healthcare NA931
Anti-rabbit-HRP	Donkey polyclonal	WB (1:5000)	GE-Healthcare NA934
Anti-mouse AF488	Goat polyclonal	IF (1:200)	Invitrogen A11029
Anti-rabbit AF594	Goat polyclonal	IF (1:200)	Invitrogen A11012
Anti-rat AF568	Goat polyclonal	IF (1:200)	Invitrogen A11077

*WB, western blot; IF, immunofluorescence; ChIP-seq, chromatin immunoprecipitation-sequencing

Ontoso, D., I. Acosta, F. van Leeuwen, R. Freire, and P.A. San-Segundo. 2013. Dot1-dependent histone H3K79 methylation promotes activation of the Mek1 meiotic checkpoint effector kinase by regulating the Hop1 adaptor. *PLoS Genet*. 9:e1003262.
